# 

**Published:** 1876-03

**Authors:** 


CONTENTS.

| | Page | | Page |
|---|---|---|---|
| Leguminous Food | 97 | Chronic Nasal Catarrh | 122 |
| Honesty and Push | 98 | Counterfeits | 124 |
| Cancer | 99 | Body and Mind | 125 |
| Do you Mean what you Say? | 100 | How it is Done | 126 |
| Medicines | 101 | In the Twilight | 128 |
| Dentifrice | 103 | Abuse of Purgative Medicines | 129 |
| Groundless Fears | 104 | Households Contrasted | 132 |
| Scarlet Fever | 105 | Cold Philosophy | 134 |
| Comfort for Suffering Feet | 107 | Public School Children | 137 |
| The Services of Chemistry to Pharmacy | 108 | Coffee Drinking | 141 |
| The Health Food Company | 109 | Surgical Instruments | 143 |
| Curative Power of Hot Springs | 120 | Burying Alive | 143 |
| | | Sedentary Habits | 144 |

